# Kinetostatic Modeling and Performance Analysis of a Symmetric Redundant-Actuated 4-PSS Compliant Parallel Micro-Motion Mechanism

**DOI:** 10.3390/mi17040439

**Published:** 2026-03-31

**Authors:** Jun Ren, Yahao Lu

**Affiliations:** Hubei Key Laboratory of Modern Manufacturing Quantity Engineering, School of Mechanical Engineering, Hubei University of Technology, Wuhan 430068, China; hbutlyh@163.com

**Keywords:** 4-pss, compliant parallel mechanism, kinetostatic modeling, redundant actuation, spatial three-translation, parasitic motion

## Abstract

A symmetric redundant-actuated 4-PSS compliant parallel micro-motion mechanism is proposed to meet the high requirements for stiffness and motion precision in micro-nano manipulation. First, the screw theory is employed to confirm that the mechanism possesses spatial three translational (3T) degrees of freedom along the X, Y and Z axes. On this basis, the global compliance model of the mechanism is constructed by combining the compliance matrix method with coordinate transformation technology, and the kinetostatic model reflecting the mapping relationship between input force/displacement and output displacement is further derived. The finite element analysis (FEA) is used to verify the kinetostatic model, and the results show that under the predefined spiral trajectory, the maximum absolute error between the theoretical calculation and the simulation result is less than 6 × 10^−7^ m, which proves the high accuracy of the established model. Moreover, a comprehensive performance analysis of the 4-PSS mechanism is carried out from the perspectives of output stiffness and parasitic motion, with the traditional 3-PSS compliant parallel mechanism as the reference. The comparative results indicate that within the specified 50 μm cubic workspace, the 4-PSS mechanism achieves a 33.3% improvement in output stiffness and a 28.15% reduction in the maximum parasitic displacement compared with the 3-PSS mechanism, while maintaining excellent global stiffness isotropy (*GSI*). Sensitivity analysis confirms the robustness of these advantages against manufacturing variations, and the workspace-to-footprint ratio remains unchanged. This research verifies that the introduction of symmetric redundant actuation branch chains can effectively enhance the comprehensive performance of compliant parallel micro-motion mechanisms and provide engineering references for the redundant design and performance optimization of high-precision compliant parallel mechanisms in the field of micro-nano manipulation.

## 1. Introduction

As precision actuators achieving micro/nano-scale positioning through elastic deformation, compliant micro-motion stages have become a core research subject in high-precision technology, garnering extensive attention from both academia and industry due to their inherent characteristics of being backlash-free, frictionless, and capable of smooth, high-precision motion. In the biomedical sector, such stages are widely applied in fields such as biological cell manipulation [1,2,3,4] and minimally invasive surgery [5,6,7,8]. In precision manufacturing, these platforms primarily facilitate applications like precision machining and microelectronics assembly [9,10,11,12,13,14]. As positioning accuracy requirements in these fields advance from the micron to the nanometer scale, more stringent demands are imposed on key performance metrics including motion accuracy, load capacity and output stability. Accordingly, research into core technologies such as kinematic modeling, precision enhancement and stiffness improvement bears considerable theoretical and practical significance.

The kinetostatic model of a compliant mechanism is core to characterizing the force or displacement transmission relationship between the input and output; furthermore, it serves as the theoretical foundation for assessing and optimizing the mechanism’s comprehensive performance. Yang et al. [15] conducted a static analysis on a 3-RRRP fully compliant parallel micro-motion mechanism, demonstrating that structural modifications could effectively enhance its static performance. Kong et al. [16] designed a 3-PRS precision compliant parallel platform with large-stroke flexure spherical and revolute joints. The kinematic model indicated that the platform achieved a large motion range with a linear displacement of over 20 mm along the Z axis and an angular displacement of approximately 18° around the X and Y axes. Lu et al. [17] proposed a novel 6-DOF 3-UPS multi-fingered parallel manipulator and systematically analyzed the active actuation force/torque and workspace characteristics based on the mechanism’s static model. More recently, Ren et al. [18,19,20] derived kinetostatic models for mechanisms such as the 3-PSS, 4-PSS&S, and n-4R. Using these models, they analyzed how workspace morphology, parasitic displacement amplitude, and output stiffness characteristics evolve with geometric parameters, providing explicit theoretical guidance for structural optimization. Synthesizing the aforementioned theoretical models and empirical cases, it is evident that kinetostatic modeling and analysis constitute an indispensable and critical step in the design and optimization of compliant micro-motion stages.

In the research of parallel mechanisms, redundant actuation limbs are frequently introduced to enhance kinematic performance, such as optimizing force transmission characteristics, eliminating singularities, and improving output accuracy and stability [21,22,23,24,25,26]. Kim et al. [21] proposed the 3-SPS/S redundant motion mechanism, confirming that the elimination of unnecessary degrees of freedom in the configuration space can realize kinematic redundancy, reduce the link displacement by up to 36% via optimal solution solving, and expand the mechanism workspace to 96.8% by avoiding geometric singularities through conformal geometric algebra. Dong et al. [22] analyzed the impact of redundant actuation on the 4-UPS&UP mechanism; through a comparison of global kinematic indices and the effective workspace, they demonstrated that the redundant configuration significantly outperforms the non-redundant counterpart in multiple performance metrics. Chai et al. [27] proposed a redundant-actuated 1T2R 2UPR-2PRU parallel robot and evaluated its performance based on stiffness indices, providing a reference for practical applications. Collectively, these studies substantiate the positive role of redundant actuation in enhancing mechanism performance. Ma et al. [28], drawing inspiration from the Delta architecture, proposed a novel redundant 6-DOF mechanism where each limb consists of a Delta parallel unit and a spherical-joint redundant link; results indicated that this configuration effectively mitigates mechanical interference risks and expands the workspace. Ren et al. [18,29] extended the application of redundant actuation from rigid to compliant parallel mechanisms, focusing on an n-4R compliant micro-positioning stage and expanding its workspace via redundancy. However, current research reveals that actuation redundancy is predominantly concentrated on rigid parallel mechanisms, with relatively few applications in compliant parallel mechanisms, suggesting significant scope for further exploration.

Motivated by the need for superior precision in micro-manipulation, this paper proposes a symmetric redundantly actuated 4-PSS compliant parallel mechanism based on the conventional 3-PSS configuration. The remainder of this paper is structured as follows: Section 2 describes the system architecture. Section 3 formulates the compliance model and derives the kinetostatic equations linking input force to output displacement, validating the theoretical framework through finite element simulations. Section 4 substantiates the advantages of the redundant 4-PSS design by comparing its kinetostatic performance—including accuracy, load capacity, and stability—with that of the non-redundant 3-PSS mechanism. Moreover, practical aspects such as manufacturing sensitivity and workspace-to-footprint ratio are evaluated to provide a comprehensive justification. Finally, Section 5 offers concluding remarks.

## 2. Description of the 4-PSS Compliant Parallel Micro-Motion Mechanism

**Figure 1:**
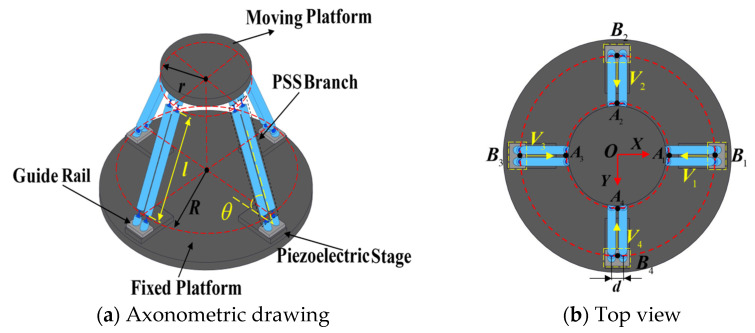
4-PSS mechanism structure diagram.

The structure of the symmetric redundant-actuated 4-PSS compliant parallel stage is shown in Figure 1. It consists of a moving platform, a fixed base, and four symmetrically arranged PSS branch chains (90° spacing), actuated by piezoelectric stages via guide rails. Each branch chain has two identical parallel links, connecting the slider to the moving platform through a compliant spherical hinge (*S*). According to screw theory and the modified Kutzbach–Grübler formula, the mechanism has 3-DOF translational mobility (3T) in the *X*, *Y*, and *Z* directions, identical to the 3-PSS mechanism. Points *A*_i_ and *B*_i_ (*i* = 1, 2, 3, 4) represent the midpoint of the center line of the compliant spherical hinges at the link ends. The geometric parameters are defined as follows: *r* and *R* are the radii of the circles formed by *A*_i_ and *B*_i_, respectively; *l* is the link length (distance between *A*_i_ and *B*_i_); *d* is the horizontal distance between adjacent parallel links; and *θ* is the initial angle between the link and the base.

## 3. Kinetostatic Modeling of a 4-PSS Compliant Parallel Micro-Motion Platform

In this section, the global compliance model of the mechanism is first established based on the compliance matrix method (CMM). The mechanism is then simplified into an equivalent spring system to derive the governing equations of elastic deformation. Based on these equations, a kinetostatic model mapping input force to output displacement is formulated. Finally, the validity of the proposed model is verified by comparing theoretical calculations with ANSYS simulation results along a specified spherical trajectory.

### 3.1. Compliance Modeling

The compliance model is the core mathematical basis for characterizing the force–displacement transfer characteristics of compliant mechanisms. To accurately analyze the kinetostatic behavior of the symmetric redundant-actuated 4-PSS mechanism, its overall compliance matrix must be established first. This can not only reveal the mapping relationship between input loads and end-effector pose but also provide the necessary theoretical support for the subsequent evaluation of stiffness performance.

#### Compliance Modeling of Each Branch Chain and the Overall Mechanism

**Figure 2:**
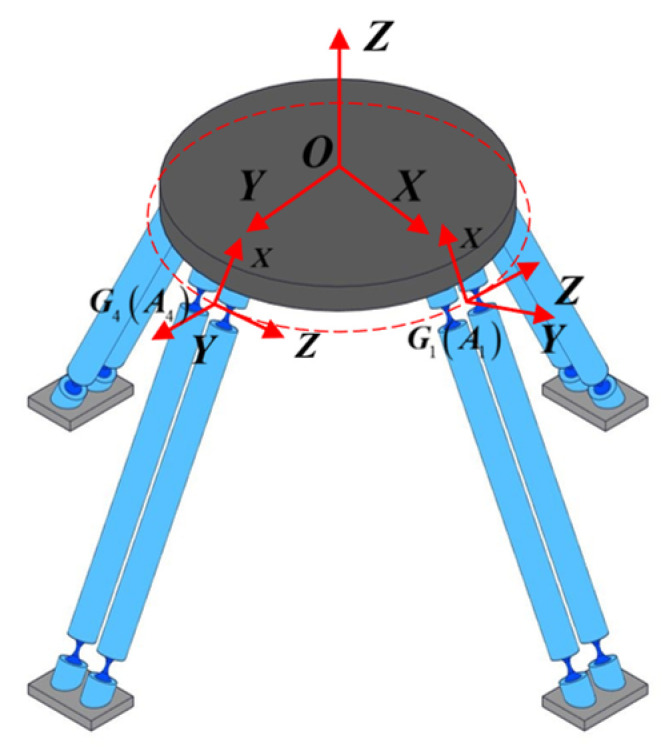
4-PSS structure branch chain coordinate diagram.

Since the mechanism is composed of four PSS branch chains with circumferential symmetric distribution, its overall compliance modeling can follow the derivation logic of “from single-branch chain analysis to overall synthesis”. The specific steps are as follows: first, establish the compliance models of the PSS branch chain, then derive the overall compliance matrix of the mechanism by using the coordinate transformation and the principle of parallel superposition. The global coordinate system {*O-XYZ*} (defined as {*O*} below) is located at the central point *O* on the upper surface of the moving platform, with its X and Y axes pointing to *OA*_1_ and *OA*_4_ respectively (shown in Figure 2). A local coordinate system {*G_i_-XYZ*} for each branch chain is established at the midpoint of the connecting line between the centers of the two compliant spherical hinges connected to the moving platform. Since the theory of such compliance modeling has been elaborated in detail in reference [20], the derivation details are not repeated in this paper, and the core conclusions and calculation formulas are given directly. The compliance matrix of the PSS1 branch chain can be calculated by the following formula:

(1) $$C_{1}=\sum_{i=1}^{2}C_{1,i}$$

where ***C***_1_ and ***C***_1,i_ denote the compliance matrices of branch 1 and the compliant spherical hinge ***S***_1,i_ in the global coordinate system {*O*}, respectively, and *i* = 1,2 represents the number of compliant spherical hinges in the branch chain. The specific calculation of the compliance matrix for the compliant spherical hinge ***C***_1,i_ is given in Appendix A. By further applying the principle of parallel stiffness superposition, the expression for the compliance matrix of the PSS1 branch chain is as follows:

(2) $$C_{PSS1}={\left(\sum_{i=1}^{2}C_{i}^{-1}\right)}^{-1}$$

To establish the overall compliance model of the mechanism, the compliance of the PSS1 branch chain is taken as an example. Since the four PSS branch chains of the mechanism are uniformly distributed, the compliance matrix of the PSS1 branch chain obtained from Equation (2) is rotated by 90°, 180° and 270° around the *Z*-axis of {*O*}. Thus, the compliance matrices of the other three PSS branch chains in the global coordinate system {*O*} can be derived. The calculation formulas are given as follows:

(3) $$C_{PSSi}=\left[T_{R,\pi \left(i-1\right)/2}\right]C_{PSS1}{\left(\left[T_{R,\pi \left(i-1\right)/2}\right]\right)}^{T}$$

where ***T***_*R*,*π*(i − 1)/2_ denotes the rotation matrix for coordinate transformation:

$$\left[T_{R,\pi \left(i-1\right)/2}\right]=\left[\begin{array}{cc} R_{z,\pi \left(i-1\right)/2} & 0_{3\times 3}\\ 0_{3\times 3} & R_{z,\pi \left(i-1\right)/2} \end{array}\right]$$

In summary, the overall compliance matrix ***C***_*Total*-4_ of the redundant-actuated 4-PSS compliant parallel micro-positioning platform in the global coordinate system {*O*} is derived based on the principle of parallel stiffness superposition, which is expressed as follows:

(4) $$C_{Total-4}={\left(\sum_{i=1}^{4}C_{PSSi}^{-1}\right)}^{-1}$$

### 3.2. Kinetostatic Modeling

The 4-PSS compliance model established previously describes the force–displacement relationship at the central point of the moving platform. However, the actual motion of the compliant parallel mechanism is achieved by applying actuating forces via sliders. Therefore, to reveal the force and displacement transmission mechanism between the slider driving input and the output at the central point of the moving platform, a kinetostatic mapping model needs to be established. For this reason, this paper refers to the modeling idea in reference [18] and constructs the kinetostatic model of the 4-PSS mechanism with slider driving forces as input variables.

**Figure 3:**
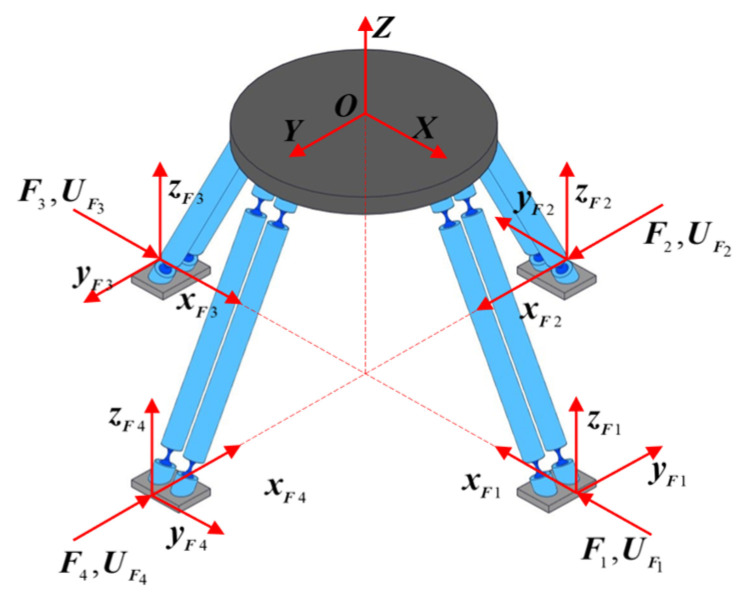
4-PSS mechanism force coordinate.

In this section, the global coordinate system {*O*} is consistent with that used in compliance modeling in the previous section. The superposition principle is adopted to derive the model for the mechanism’s input forces and output displacements. The concept of equivalent stiffness is introduced, and each PSS branch chain is equivalent to a stiffness element with stiffness ***K***_PSSi_. The force coordinate system of the mechanism is shown in Figure 3. A force coordinate system *F_i_*-*x_i_y_i_z_i_* (*i* = 1, 2, 3, 4) is established at the central point of the bottom end face of each of the four sliders. The corresponding generalized input force is defined as ***F****_i_* = [*m*_x_,*m*_y_,*m*_z_,*f*_x_,*f*_y_,*f*_z_]^T^. The displacement of the central point *O* of the moving platform in the global coordinate system {*O*} induced by the input force ***F****_i_* is denoted as ***U****_O_* = [*θ*_x_,*θ*_y_,*θ*_z_,*δ*_x_,*δ*_y_,*δ*_z_]^T^. When the four input forces ***F****_i_*(*i* = 1, 2, 3, 4) are applied simultaneously along their respective force coordinate systems {*F*_i_}, the total output displacement of the end platform is obtained as ***U****_all_* by applying the superposition principle.

**Figure 4:**
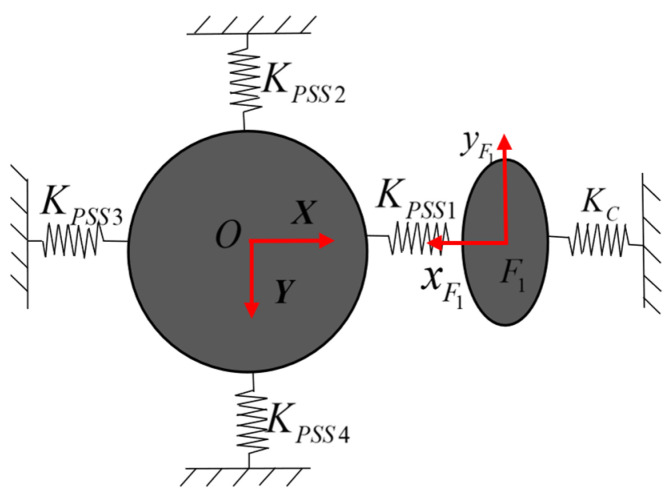
Equivalent spring system model under *F*_1_.

When a piezoelectric actuator is actuated, the slider moves freely along the guide rail direction and is strictly constrained in the other five non-functional directions. To express this physical constraint in mathematical form in the stiffness model, it is assumed that the slider consists of a 6-DOF virtual spring system and the stiffness in the non-moving directions is much higher than that in the moving direction. A rigid simplification is adopted in the theoretical model to obtain the constraint stiffness matrix ***K****_C_* = *diag*(*K_θx_*,*K_θy_,K_θz_*,*K_δx_*,*K_δy_*,*K_δz_*). Considering the practical situation, the stiffness *K_θx_* along the functional movement direction of the guide rail is infinitesimal, while the other five stiffness values are infinite. Taking the single action of the input force ***F***_1_ as an example, the equivalent spring system model of the mechanism is shown in Figure 4. According to Hooke’s Law, the governing equation for the elastic deformation of the spring system can be expressed as

(5) $$\left[\begin{array}{cc} {\left(K_{OO}\right)}_{F1} & K_{OF1}\\ K_{F1O} & K_{F1F1} \end{array}\right]\left[\begin{array}{c} U_{1}\\ U_{F1} \end{array}\right]=\left[\begin{array}{c} F_{O}\\ F_{1} \end{array}\right]$$

where ***U***_1_ denotes the output displacement of the midpoint of the moving platform in the global coordinate system {*O*} when force *F*_1_ acts alone. ***U****_F_*_1_ represents the displacement of the slider in the force coordinate system *F*_1_-*x*_1_*y*_1_*z*_1_. ***F****_O_* is the force applied at the central point of the moving platform. The stiffness matrices (***K****_OO_*)*_F_*_1_, ***K****_OF_*_1_, ***K****_F_*_1*O*_ and ***K****_F_*_1*F*1_ are calculated as follows:

(6) $$\left\{\begin{array}{l} {\left(K_{OO}\right)}_{F1}=K_{PSS1}+K_{PSS2}+K_{PSS3}+K_{PSS4}\\ K_{F1F1}=K_{PSS1}^{F1}+K_{C}\\ K_{OF1}=-K_{PSS1}^{O,F1}\\ K_{F1O}=-K_{PSS1}^{F1,O} \end{array}\right.$$

where the superscripts *F*_1_ and *O* denote the coordinate systems corresponding to the stiffness matrices. Specifically, the double-superscript stiffness matrix $K_{PSS1}^{O,F1}$ indicates that the corresponding force and displacement are defined in the coordinate systems {*F*_1_} and {*O*}, respectively. The same definition applies to the stiffness matrix $K_{PSS1}^{F1,O}$. ***K****_PSS_*_1_, ***K****_PSS_*_2_, ***K****_PSS_*_3_ and ***K****_PSS_*_4_ denote the equivalent stiffnesses of the four PSS branch chains with respect to the global coordinate system {*O*}, respectively. The specific calculation formulas are given as follows:

(7) $$K_{PSSi}={\left(C_{PSSi}\right)}^{-1}$$

***K****_F_*_1*F*1_, ***K****_OF_*_1_ and ***K****_F_*_1*O*_ are determined via matrix transformation:

(8) $$\left\{\begin{array}{l} K_{F1F1}={\left({\left[T\right]}_{G_{1}}^{F_{1}}\right)}^{-T}\left[K_{PSS1}^{G_{1}}\right]{\left({\left[T\right]}_{G_{1}}^{F_{1}}\right)}^{-1}+K_{C}\\ K_{OF1}=-{\left({\left[T\right]}_{G_{1}}^{O}\right)}^{-T}\left[K_{PSS1}^{G_{1}}\right]{\left({\left[T\right]}_{G_{1}}^{F_{1}}\right)}^{-1}\\ K_{F1O}=-{\left({\left[T\right]}_{G_{1}}^{F_{1}}\right)}^{-T}\left[K_{PSS1}^{G_{1}}\right]{\left({\left[T\right]}_{G_{1}}^{O}\right)}^{-1} \end{array}\right.$$

where [***T***${]}_{G_{1}}^{F_{1}}$ denotes the adjoint matrix of the local branch chain coordinate system {G1} with respect to the force coordinate system {*F*_1_}. [***T***${]}_{G_{1}}^{O}$ denotes the adjoint matrix of the local branch chain coordinate system {*G*_1_} with respect to the global coordinate system {*O*}. Detailed calculations are referred to Appendix B. The stiffness matrix $K_{PSS1}^{G_{1}}$ is derived from the compliance matrix in the local branch chain coordinate system.

(9) $$K_{PSS1}^{G_{1}}={\left(C^{G_{1}}\right)}^{-1}$$

Considering the constrained state of the 4-PSS mechanism without external force, where ***F****_O_* = 0, it can be derived from Equation (5) that

(10) $$\left\{\begin{array}{l} U_{1}=\underset{C_{F1}}{\underset{︸}{-{\left({\left(K_{OO}\right)}_{F1}-K_{OF1}K_{F1F1}^{-1}K_{F1O}\right)}^{-1}\left(K_{OF1}K_{F1F1}^{-1}\right)}}\cdot F_{1}\;\\ U_{F1}={\left(\left(K_{F1F1}\right)-K_{F1O}{\left(K_{OO}\right)}_{F1}^{-1}K_{OF1}\right)}^{-1}\cdot F_{1} \end{array}\right.$$

Equation (10) describes the mapping relationship between the input force ***F***_1_ and the input displacement ***U****_F_*_1_, where ***C****_F_*_1_ is the mapping stiffness matrix relating ***F***_1_ and ***U***_1_. Similarly, the mapping matrix between the input force ***F***_1_ and the output displacement ***U****_F_*_1_ can be derived. Likewise, when the input force ***F***_i_ (*i* = 1, 2, 3, 4) acts independently, the output displacement of the moving platform can be expressed as

(11) $$U_{i}=\underset{C_{Fi}}{\underset{︸}{-{\left({\left(K_{OO}\right)}_{Fi}-K_{OFi}K_{FiFi}^{-1}K_{FiO}\right)}^{-1}\left(K_{OFi}K_{FiFi}^{-1}\right)}}\cdot F_{i}\left(i=1,2,3,4\right)$$

Considering the 90° circumferential symmetric distribution of the mechanism branch chains, the corresponding matrices of the other three branch chains are obtained via rotation matrices.

(12) $$\left\{\begin{array}{l} {\left(K_{OO}\right)}_{F_{i}}=[T_{R,(i-1)\pi /2}]\left[{\left(K_{OO}\right)}_{F1}\right]{\left([T_{R,(i-1)\pi /2}]\right)}^{T}\\ K_{OFi}=[T_{R,(i-1)\pi /2}]\left[K_{OF1}\right]\\ K_{FiO}=\left[K_{F1O}\right]{\left([T_{R,(i-1)\pi /2}]\right)}^{T}\\ K_{FiFi}=K_{F1F1}(i=1,2,3,4) \end{array}\right.$$

Based on the superposition principle and combined with Equation (12), the output displacements corresponding to the four input forces ***F****_i_* are superimposed. The total output displacement ***U***_all_ of the central point of the moving platform in the global coordinate system {*O*} is derived, and its expression is given as follows:

(13) $$U_{all}=\sum_{i=1}^{4}U_{i}=\left[\begin{array}{cccc} C_{F1} & C_{F2} & C_{F3} & C_{F4} \end{array}\right]\cdot \left[\begin{array}{c} F_{1}\\ F_{2}\\ F_{3}\\ F_{4} \end{array}\right]$$

For practical engineering applications, the input forces ***F***_i_ of the sliders along the driving directions and the displacement components ***δ****_all_* = [*δ_x_*,*δ_y_*,*δ_z_*]*^T^* of the moving platform in three translational DOFs are further extracted from Equation (13). Thus, the input force–output displacement model of the redundant-actuated 4-PSS compliant parallel mechanism can be expressed as

(14) $$\left[\begin{array}{c} \delta_{X}\\ \delta_{Y}\\ \delta_{Z} \end{array}\right]=\left[\begin{array}{cccc} {\left(C_{F1}\right)}_{\left[4-6,4\right]} & {\left(C_{F2}\right)}_{\left[4-6,4\right]} & {\left(C_{F3}\right)}_{\left[4-6,4\right]} & {\left(C_{F4}\right)}_{\left[4-6,4\right]} \end{array}\right]\cdot \left[\begin{array}{c} f_{1}\\ f_{2}\\ f_{3}\\ f_{4} \end{array}\right]$$

where (***C***_*F*i_)_[4-6,4]_(*i* = 1, 2, 3, 4) denotes the last three elements of the fourth column of the mapping matrix ***C****_F_*_i_(*i* = 1, 2, 3, 4).

In practice, considering the displacement output characteristics of piezoelectric actuators, the input displacements of the sliders are easier to obtain than the input driving forces. Therefore, to match practical application scenarios, the kinetostatic equation of input displacement to output displacement is further derived based on Equation (14). A more direct motion transmission model is thus established. Assume the input displacement of the slider is ***U****_Fi_* = [0,0,0,*δ_i_*,0,0]*^T^*. Combining Equations (10) and (13), the expression relating the input displacement ***U****_F_*_i_ and the output displacement ***U****_all_* can be obtained as follows:

(15) $$U_{all}=\sum_{i=1}^{4}U_{i}=\left[\begin{array}{cccc} C_{U1} & C_{U2} & C_{U3} & C_{U4} \end{array}\right]\cdot \left[\begin{array}{c} U_{F1}\\ U_{F2}\\ U_{F3}\\ U_{F4} \end{array}\right]$$

where ***C****_U_*_i_ is the input displacement-to-output displacement mapping matrix of the mechanism, whose expression is given as follows:

(16) $$C_{Ui}=\underset{C_{Fi}}{\underset{︸}{-{\left({\left(K_{OO}\right)}_{Fi}-K_{OFi}K_{FiFi}^{-1}K_{FiO}\right)}^{-1}\left(K_{OFi}K_{FiFi}^{-1}\right)}}\left(\left(K_{FiFi}\right)-K_{FiO}{\left(K_{OO}\right)}_{Fi}^{-1}K_{OFi}\right)$$

Based on the superposition principle, the input displacement-to-output displacement model of the symmetrically redundant-actuated 4-PSS compliant parallel micro-motion platform is given by

(17) $$\left[\begin{array}{c} \delta_{X}\\ \delta_{Y}\\ \delta_{Z} \end{array}\right]=\left[\begin{array}{cccc} {\left(C_{U1}\right)}_{\left[4-6,4\right]} & {\left(C_{U2}\right)}_{\left[4-6,4\right]} & {\left(C_{U3}\right)}_{\left[4-6,4\right]} & {\left(C_{U4}\right)}_{\left[4-6,4\right]} \end{array}\right]\cdot \left[\begin{array}{c} \delta_{1}\\ \delta_{2}\\ \delta_{3}\\ \delta_{4} \end{array}\right]$$

### 3.3. Model Verification

In this paper, ANSYS Workbench 2023 R1 is adopted. The effectiveness of the displacement–displacement mapping model of the 4-PSS mechanism established above is verified via its static structural module. The geometric dimensions of the simulation model are strictly consistent with those in the theoretical derivation (shown in Table 1). For the compliant spherical hinge, beryllium copper (CuBe) is selected as the material, with key properties including Young’s modulus *E*, density *ρ*, and Poisson’s ratio *ν*. The cutting radius of the hinge is denoted as *R*_2_, and its minimum thickness is *t*. All these parameters are listed in Table 1. The other rigid components of the mechanism adopt the material properties of structural steel, which are also summarized in Table 1. Regional mesh refinement is applied to the mechanism to ensure calculation accuracy. Given the complexity of its geometric curved surface and the demand for a high-precision deformation solution, the compliant spherical hinge is meshed with tetrahedral elements of a 0.1 mm element size. A preliminary mesh independence study confirmed that reducing the mesh resolution from 0.5 mm to 0.1 mm resulted in less than a 5% change in the simulated trajectory, thereby validating the adequacy of the 0.1 mm mesh. The remaining rigid components are automatically meshed with tetrahedral elements by the software.

The specific verification procedures are as follows: Firstly, the predefined target trajectory is discretized into multiple sampling points. Secondly, the coordinates of the sampling points are substituted into Equation (17) of the theoretical model, and the corresponding theoretical input displacements are solved using the pseudo-inverse algorithm. Subsequently, this set of theoretical input displacements is applied as loads to the input end of the finite element model, and the simulated pose trajectory of the 4-PSS mechanism is obtained. Finally, the accuracy of the theoretical model is quantitatively verified by comparing the deviation between the simulated trajectory and the predefined target trajectory.

The predefined target trajectory of the moving platform is given as follows:

(18) $$\left\{\begin{array}{l} x=L_{1}\sin\gamma \cos p\gamma \\ y=L_{1}\sin\gamma \sin p\gamma \\ z=L_{1}\cos\gamma \\ L_{1}=1\times 10^{-4}\left(m\right)\;,\;0\leq \gamma \leq \pi ,\;p=10 \end{array}\right.$$

**Figure 5:**
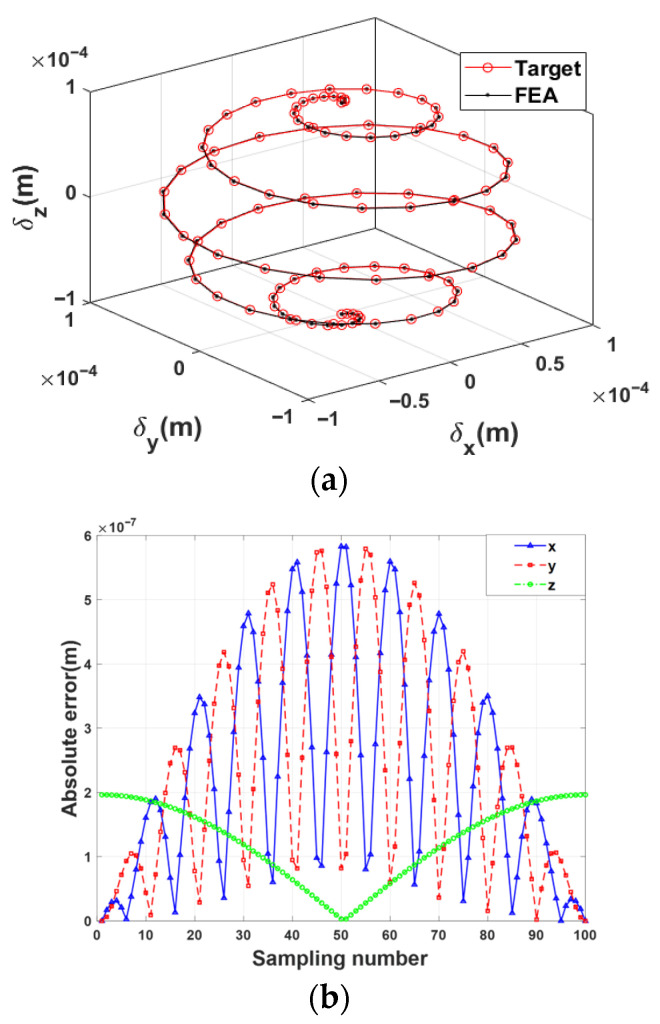
(**a**) Trajectory comparison diagram. (**b**) Absolute displacement error of trajectories.

The comparison results of the 3D spatial trajectory and the absolute displacement errors along each axis are shown in Figure 5a,b, respectively. They show that the simulated trajectory and the predefined target trajectory show a high degree of spatial consistency. Further quantitative analysis indicates that the maximum absolute displacement error of the mechanism in the three translational directions is within 6 × 10^−7^ m. Within the allowable error range, this result validates the correctness and reliability of the static model established in this paper.

## 4. Kinetostatic Performance Analysis of the Mechanism

To comprehensively and quantitatively evaluate the enhancement effect of symmetric redundant actuation on the overall performance of the 4-PSS compliant parallel micro-motion platform, a multi-dimensional performance evaluation framework is established in this section. The framework incorporates output stiffness (load-carrying capacity), stiffness condition number (isotropy), and parasitic motion (motion accuracy). Considering that evaluation at a single equilibrium position cannot truly reflect global performance characteristics, a task workspace is defined. By comparing the performance distribution nephograms at specific sections of this workspace, the spatial variation gradient and distribution uniformity of each performance index are intuitively revealed. On this basis, an in-depth and comprehensive comparison is carried out between the redundant-actuated 4-PSS compliant parallel micro-motion platform and the conventional 3-PSS compliant parallel micro-motion platform.

### 4.1. Performance Evaluation of Kinetostatic Characteristics of the Mechanism

**Figure 6:**
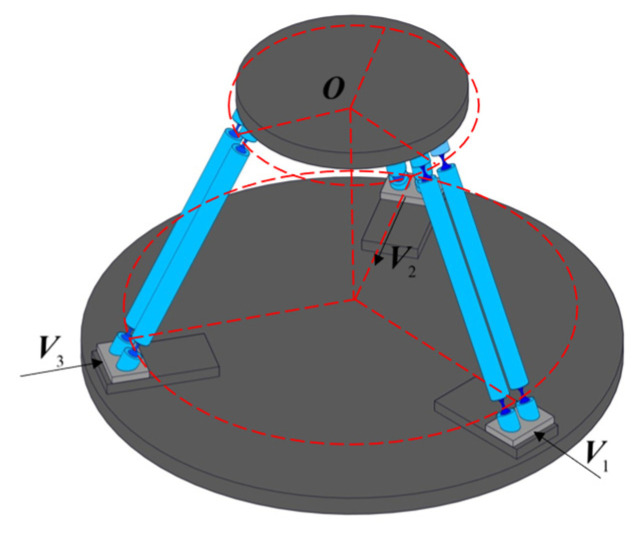
Schematic diagram of the 3-PSS structure.

Within the limits of elastic deformation for compliant spherical hinges and the allowable stress of the material, and combined with the structural characteristics of the 4-PSS compliant parallel micro-motion mechanism, its task workspace *W_t_* is defined as a cube with a side length of 50 μm. Taking the 3-PSS compliant parallel micro-motion platform as the reference object (shown in Figure 6), the kinetostatic performances of the proposed 4-PSS compliant parallel micro-motion platform and the 3-PSS one are compared and analyzed under the same geometric dimensions and task workspace *W_t_*. The distribution of each performance index for the two mechanisms in the section *Z* = *H_t_*/2 within the task workspace *W_t_* is investigated.

#### 4.1.1. Output Stiffness

In precision positioning and micro-nano manipulation (e.g., photolithography alignment and biological cell penetration), the high demand for high load-carrying capacity requires a rigorous evaluation of the mechanism’s deformation resistance. The essential difference between compliant parallel mechanisms and conventional rigid mechanisms is that the former realizes motion transmission by means of the elastic deformation of compliant elements. However, this transmission manner inevitably induces elastic displacement at the end-effector under external loads, which directly constrains the structural stability of the mechanism. In this context, a thorough investigation into the output stiffness of the mechanism is critical. This index is not only a key measure to characterize the resistance to load-induced deformation but also a core indicator that determines the load-carrying performance and structural stability.

Based on the global compliance matrix of the mechanism derived from Equation (4), the output stiffness matrices of the two configurations can be obtained according to the reciprocal relationship between stiffness and compliance:

(19) $$\left\{\begin{array}{l} K_{output-4}={\left(C_{Total-4}\right)}^{-1}\\ K_{output-3}={\left(C_{Total-3}\right)}^{-1} \end{array}\right.$$

**Figure 7:**
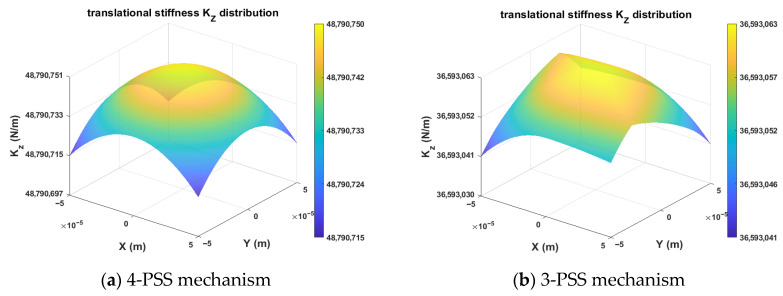
Contour distribution of *K_Z_* cross-sections on *W_t_*.

For the 4-PSS and 3-PSS mechanisms with identical motion characteristics (3T), the Z-direction translational stiffness *K_Z_* is selected for comparative analysis (shown in Figure 7). The results show that the peak stiffness of the 4-PSS mechanism is approximately 4.88 × 10^7^ N/m, which is significantly higher than the 3.66 × 10^7^ N/m of the 3-PSS mechanism, and it presents excellent central symmetric distribution. Although the stiffness of both mechanisms decays monotonically with the increase in radial distance, the 4-PSS mechanism remarkably raises the baseline of axial stiffness by virtue of redundant branch chains. This indicates that the redundant configuration achieves a substantial enhancement in load-carrying capacity.

#### 4.1.2. Stiffness Condition Number

However, a simple comparison of output stiffness magnitude only reflects the load-carrying capacity of the mechanism and cannot reveal the uniformity and numerical stability of its mechanical performance over the task workspace. Considering the characteristic that parallel mechanisms exhibit stiffness differences in different directions (i.e., anisotropy), the condition number of the stiffness matrix is introduced as a key index to evaluate the performance uniformity and stability margin of the force–displacement mapping of parallel mechanisms [30]. This index is defined as the ratio of the maximum singular value to the minimum singular value of the stiffness matrix and reflects the dispersion degree of the eigenvalues of the stiffness matrix at different poses within the task workspace. Its expression is given as follows:

(20) $$KSI=\frac{\sigma_{\max}\left(K\right)}{\sigma_{\min}\left(K\right)}$$

where *KSI* denotes the stiffness matrix condition number of the mechanism. Considering the actual motion characteristics (3T) of both mechanisms, the translational stiffness ***K*** extracted from the stiffness matrix is taken as the research object, which is derived from Equation (4). *σ_max_*(***K***) and *σ_min_*(***K***) represent the maximum and minimum singular values of the stiffness matrix ***K***, respectively. The *KSI* value generally varies with different poses of the mechanism in the workspace. When *KSI* = 1, the mechanism is isotropic at the corresponding pose with optimal transmission performance. The closer the *KSI* value is to 1, the more uniform the mechanical performance distribution of the mechanism. Conversely, a larger *KSI* indicates poorer transmission performance and a greater tendency toward singular configurations.

**Figure 8:**
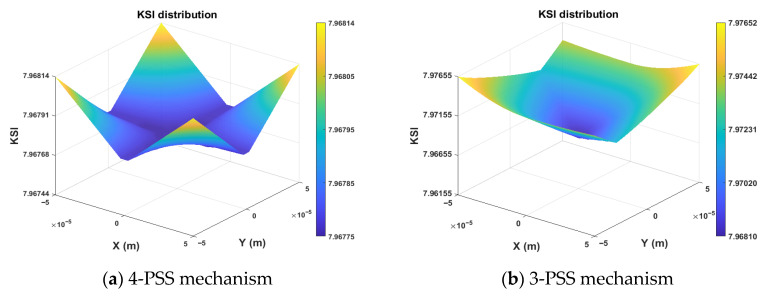
Contour distribution of *KSI* cross-sections on *W_t_*.

Figure 8 shows the distribution of the *KSI* index for the two mechanisms in the section *Z = H_t_*/2 within the task workspace *W_t_*. It can be observed that, in the same section, the *KSI* distribution of the 4-PSS mechanism is cross-shaped, and the *KSI* value in the central region is close to the minimum, indicating highly uniform and stable mechanical performance in the central area. In contrast, the 3-PSS mechanism exhibits an obvious diamond-shaped distribution, with a significant stiffness gradient difference between the diagonal directions and other directions, reflecting the anisotropy of its stiffness performance.

Considering that *KSI* only characterizes the performance of the mechanism at a specific pose and can only be used as a local stiffness index, it cannot evaluate the overall performance of the mechanism. Therefore, another global stiffness performance index *GSI* is adopted for the overall evaluation [30]. Its computational formula is given as follows:

(21) $$GSI=\frac{\int_{W_{t}}\left(KSI\right)dW}{\int_{W_{t}}dW}$$

where *W_t_* denotes the task workspace of the mechanism. In numerical computation, the discretization method is used to mesh the task workspace *W_t_*, and the global stiffness performance index (*GSI*) over *W_t_* is obtained by traversing and calculating the *KSI* values at all nodes.

Under the same task workspace (the section *Z* = *H_t_*/2), the calculated results show that the *GSI* value of the redundant-actuated 4-PSS mechanism is 7.968, which is lower than the 7.972 of the non-redundant-actuated 3-PSS mechanism. A smaller *GSI* represents a more uniform stiffness distribution. This result indicates that the addition of redundant branch chains improves the uniformity of force transmission globally to a certain extent.

In terms of distribution characteristics, the stiffness condition number contour of the 4-PSS mechanism shows a regular cross-shaped low-value region with better isotropy. Compared with the skewed distribution of the 3-PSS mechanism, this feature is more beneficial to the decoupling of control algorithms and stability design. In summary, the symmetric redundant actuation configuration fully demonstrates its design superiority: it not only breaks the trade-off between stiffness magnitude and motion dexterity in conventional parallel mechanisms but also effectively maintains or even optimizes the motion accuracy while significantly improving the system load-bearing stiffness (by approximately 33%), achieving a dual improvement of enhanced stiffness magnitude and better global isotropy.

#### 4.1.3. Parasitic Displacement

In ultra-precision scanning or micro-manufacturing works, the trajectory accuracy of the end-effector is critical, and any tiny parasitic motion can result in the failure of machining or measurement. Parasitic motion specifically refers to an undesired movement that deviates from the ideal functional direction during the actual motion of the mechanism and is the main factor limiting the accuracy of compliant mechanisms. For the 4-PSS and 3-PSS compliant parallel mechanisms, this type of motion is characterized by rotations about the *X*, *Y*, and *Z*-axes accompanying the translations of the end platform along the *X*, *Y*, and *Z* directions. Theoretically, the introduction of symmetric redundant branch chains can provide additional geometric constraints, which help to suppress these non-functional degrees of freedom.

In the following paragraphs, the actual effect of the 4-PSS configuration on suppressing parasitic motion and improving trajectory accuracy is verified through specific parasitic motion calculations. Based on the verified static equations of the mechanism, the parasitic motion of the two mechanisms can be quantitatively calculated and compared by extracting the angular displacement components from the output displacement matrix using Equation (17).

The formula for the parasitic rotation angle is given as follows:

(22) $$\theta_{para_{4}}=\left[\begin{array}{c} \theta_{X_{para}}\\ \theta_{Y_{para}}\\ \theta_{Z_{para}} \end{array}\right]=\left[\begin{array}{cccc} {\left(C_{U1}\right)}_{\left[1-3,4\right]} & {\left(C_{U2}\right)}_{\left[1-3,4\right]} & {\left(C_{U3}\right)}_{\left[1-3,4\right]} & {\left(C_{U4}\right)}_{\left[1-3,4\right]} \end{array}\right]\cdot \left[\begin{array}{c} \delta_{1}\\ \delta_{2}\\ \delta_{3}\\ \delta_{4} \end{array}\right]$$

The theoretical input displacements derived from Equations (17) and (18) are substituted into Equation (22), and the parasitic rotation angles of the mechanism during actual motion can be solved using the pseudo-inverse algorithm.

**Figure 9:**
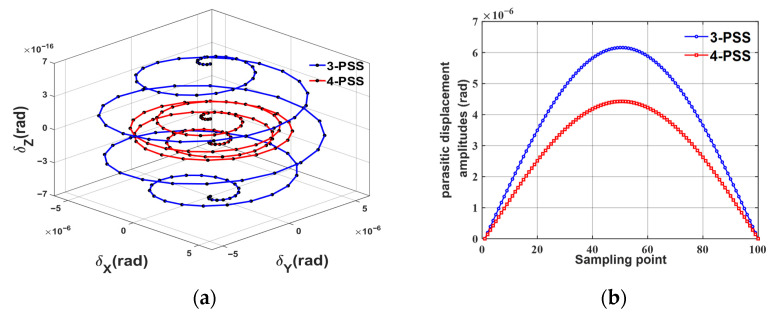
(**a**) Comparison of three-dimensional parasitic motion. (**b**) Comparison of parasitic tilt angle amplitude.

The 3D parasitic motion of the mechanism along the trajectory is shown in Figure 9a. By comparing with ***C****_F_*_i_ in Equation (16), it can be seen that the parasitic displacement component along the *Z*-axis is remarkably lower than those along the *X*-axis and *Y*-axis in order of magnitude, indicating that the mechanism has extremely high torsional stiffness in the Z-direction. Therefore, the Z -direction component is ignored in the subsequent analysis, and only the parasitic inclination angles in the *X*-*Y* plane are focused on. In view of the vector property of parasitic motion, the amplitude of the total parasitic inclination angle $\theta_{T}=\sqrt{{\theta_{X_{para}}}^{2}+{\theta_{Y_{para}}}^{2}}$ is defined as the evaluation index. The comparison results of the parasitic displacement amplitudes of the two mechanisms under the same trajectory are given in Figure 9b. The red curve representing the redundant 4-PSS mechanism is always tightly enclosed within the blue curve representing the 3-PSS mechanism. The results show that under the same task trajectory, the parasitic motion amplitude of the 4-PSS configuration is substantially suppressed, and the maximum peak value of parasitic displacement is reduced by 28.15%. This suppression effect is attributed to the over-constraint mechanism introduced by the redundant branch chains, which effectively corrects the attitude deflection of the moving platform during motion, thus significantly improving the translational motion accuracy of the micro-motion platform.

Based on the comprehensive evaluation of stiffness performance maps and parasitic motion characteristics, it can be concluded that within the preset task workspace *W_t_*, the 4-PSS redundant configuration achieves the balance between “high stiffness” and “high precision”. Specifically, this configuration increases the output stiffness magnitude by 33.3% while maintaining the stability of its global performance index (*GSI*). More importantly, its parasitic motion amplitude is substantially suppressed. This result fully confirms that the symmetric redundant actuation design not only enhances the load-carrying capacity and stability of the micro-motion platform but also significantly improves the motion guidance accuracy on the basis of maintaining the degree-of-freedom characteristics, providing theoretical data support and a reference for the optimization of high-precision micro-nano manipulation mechanisms.

**Figure 10:**
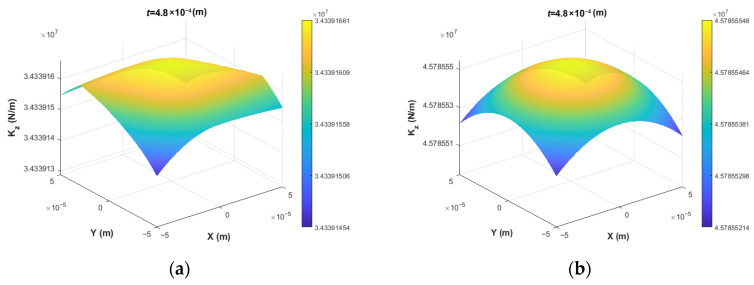

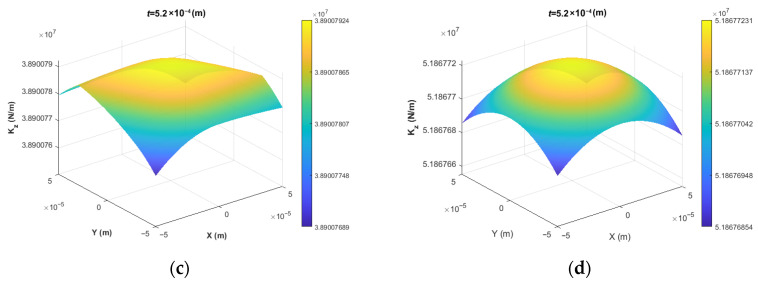
Comparison of output stiffness *K_Z_* for mechanisms at different minimum thickness of compliant spherical hinges: (**a**) 3-PSS at *t* = 0.48 mm, (**b**) 4-PSS at *t* = 0.48 mm, (**c**) 3-PSS at *t* = 0.52 mm, (**d**) 4-PSS at *t* = 0.52 mm.

**Figure 11:**
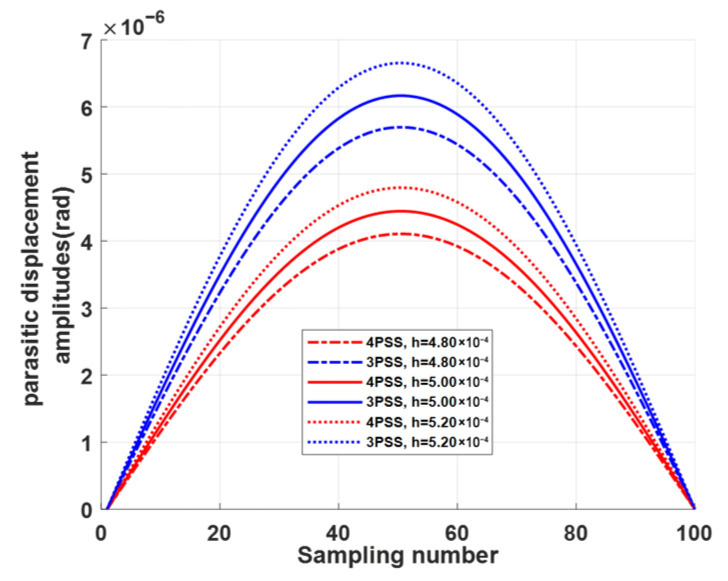
Comparison of parasitic displacement amplitudes for 3-PSS and 4-PSS mechanisms at different compliant spherical hinges.

To further verify the robustness of these conclusions, a sensitivity analysis is carried out with respect to the minimum thickness *t* of the compliant spherical hinge—the most variable geometric parameter. Varying *t* by ±4% (0.48 mm and 0.52 mm) around the nominal value *t* = 0.5 mm, the relative improvements of the 4-PSS mechanism over the 3-PSS mechanism remain stable: the stiffness enhancement stays within 33.3% (shown in Figure 10), and the reduction in maximum parasitic displacement amplitudes stays within 27.92–28.15% (shown in Figure 11). These results indicate that the superior performance of the redundant design is not sensitive to typical manufacturing variations, and the main conclusion—that the 4-PSS consistently outperforms the 3-PSS—holds firmly.

### 4.2. Engineering Value Assessment

The above comparisons confirm that the 4-PSS mechanism achieves 33.3% higher output stiffness within the same 50 μm cubic workspace, and under the same trajectory, the maximum parasitic displacement amplitude of the 4-PSS mechanism is 28.15% lower than that of the 3-PSS mechanism. While the redundant design introduces an extra branch chain and actuator—moderately increasing system cost and control complexity—a geometric analysis shows that both mechanisms can be abstracted as the frustum of a cone with identical radii and link length; thus, their theoretical footprints are the same, and the workspace-to-footprint ratio does not degrade. Considering the stringent requirements of micro/nano manipulation tasks, the moderate cost increase is justified by the substantial performance gains. This makes the redundantly actuated configuration a practical and advantageous solution for high-precision compliant parallel mechanisms.

## 5. Conclusions

This paper presents a symmetric redundant-actuated 4-PSS compliant parallel micro-motion mechanism for the high stiffness and precision demands of micro-nano manipulation. Comprehensive kinetostatic modeling, model validation and a multi-dimensional performance comparison with the conventional 3-PSS mechanism are carried out. This research clarifies the motion characteristics and prominent performance advantages of the redundantly actuated configuration, with the key conclusions summarized as follows:

1. The 4-PSS mechanism with four PSS branch chains in a 90° circumferential symmetric distribution retains the X/Y/Z three translational (3T) degrees of freedom consistent with the 3-PSS mechanism. The introduction of redundant actuation branch chains does not change the basic motion characteristics of the mechanism, realizing the performance upgrade on the basis of compatible traditional motion requirements.
2. The global compliance model and input–output kinetostatic mapping model of the 4-PSS mechanism are constructed by integrating the compliance matrix method and coordinate transformation technology. Finite element verification shows the theoretical model has high accuracy, with the maximum absolute error less than 6 × 10^−7^ m under the predefined spiral trajectory.
3. The 4-PSS mechanism shows significant comprehensive performance advantages in the same 50 μm cubic workspace: its Z-direction peak output stiffness is increased by 33.3% with a more uniform centrosymmetric distribution, the global stiffness isotropy index (GSI = 7.968) is better than that of the 3-PSS mechanism, and the maximum parasitic displacement is reduced by 28.15%. Sensitivity analysis confirms that these advantages remain stable under typical manufacturing variations. Moreover, despite the added cost and complexity of the redundant branch, the mechanism’s footprint does not increase, and the workspace-to-footprint ratio remains unchanged. Thus, the performance gains justify the engineering trade-off. Symmetric redundant actuation effectively improves the load-carrying capacity, stiffness uniformity and motion precision of the compliant parallel micro-motion mechanism and provides important theoretical and engineering references for the redundant design and performance optimization of high-precision compliant parallel mechanisms in micro-nano manipulation fields.

## Figures and Tables

**Table 1:** Geometric and material parameters of the 4-PSS compliant parallel mechanism.

Parameter	Value	Parameter	Value
*R*	58.5 mm	*E*	128 Gpa
*r*	29.7 mm	*ρ*	8000 kg/m^3^
*L*	64.3 mm	*ν*	0.3
*d*	7 mm	*t*	0.5 mm
*θ*	63.391°	*R*_2_	2.5 mm

## Data Availability

All data that support the findings of this study are included within the article.

## Appendix A

The compliance matrix of a right-circular compliant spherical hinge in the end-face center coordinate system is given as follows:

$$X=\left[\begin{array}{l} \theta_{x}\\ \theta_{y}\\ \theta_{z}\\ \delta_{x}\\ \delta_{y}\\ \delta_{z} \end{array}\right]=\left[\begin{array}{cccccc} C_{\theta_{x},m_{x}} & 0 & 0 & 0 & 0 & 0\\ 0 & C_{\theta_{y},m_{y}} & 0 & 0 & 0 & C_{\theta_{y},f_{x}}\\ 0 & 0 & C_{\theta_{z},m_{z}} & 0 & C_{\theta_{z},f_{y}} & 0\\ 0 & 0 & 0 & C_{\delta_{x},f_{x}} & 0 & 0\\ 0 & 0 & C_{\delta_{y},m_{z}} & 0 & C_{\delta_{y},f_{y}} & 0\\ 0 & C_{\delta_{z},m_{y}} & 0 & 0 & 0 & C_{\delta_{z},f_{z}} \end{array}\right]\left[\begin{array}{c} m_{x}\\ m_{y}\\ m_{z}\\ f_{x}\\ f_{y}\\ f_{z} \end{array}\right]=CF$$

The calculation formulas for each value are as follows:

$$\left\{\begin{array}{ll} C_{\theta_{x,1}m_{x}} & =\frac{64(1+v)}{\pi E}\int_{0}^{2r_{0}}\frac{1}{t{\left(x\right)}^{4}}dx,\\ C_{\theta_{y,1}m_{y}} & =C_{\theta_{z,1}m_{z}}=\frac{64}{\pi E}\int_{0}^{2r_{0}}\frac{1}{t{\left(x\right)}^{4}}dx,\\ C_{\delta_{z,1}m_{y}} & =C_{\theta_{y,1}f_{z}}=-C_{\theta_{z,1}f_{y}}=-C_{\delta_{y,1}m_{z}}=-\frac{64r_{0}}{\pi E}\int_{0}^{2r_{0}}\frac{1}{t{\left(x\right)}^{4}}dx,\\ C_{\delta_{x,1}f_{x}} & =\frac{4}{\pi E}\int_{0}^{2r_{0}}\frac{1}{t{\left(x\right)}^{2}}dx,\\ C_{\delta_{y,1}f_{y}} & =C_{\delta_{z,1}f_{z}}=\frac{64}{\pi E}\int_{0}^{2r_{0}}\frac{x^{2}}{t{\left(x\right)}^{4}}dx+\frac{k(1+v)}{\pi E}\int_{0}^{2r_{0}}\frac{1}{t{\left(x\right)}^{2}}dx \end{array}\right.$$

In the equation, *E* and *ν* represent the Young’s modulus and Poisson’s ratio of the compliant ball-and-socket joint material, respectively. *k* denotes the shear shape coefficient, with *k* = 10/9. *t*(*x*) is the thickness function of the compliant ball-and-socket joint along the *x*-axis, defined as follows:

$$t(x)=t_{\min}+2(r_{0}-\sqrt{x(2r_{0}-x)}),\;x\in [0,2r_{0}]$$

## Appendix B

The expression for transforming a matrix from coordinate system {***C****_OA_*} to coordinate system {***C****_OB_*} is

$$C_{O_{B}}={\left[T\right]}_{O_{A}}^{O_{B}}C_{O_{A}}{\left({\left[T\right]}_{O_{A}}^{O_{B}}\right)}^{T}$$

where ${\left[T\right]}_{O_{A}}^{O_{B}}$ is the coordinate-accompanying matrix, expressed as

$${\left[T\right]}_{O_{A}}^{O_{B}}=\left[\begin{array}{cc} {\left[R\right]}_{O_{A}}^{O_{B}} & 0_{3\times 3}\\ {\left[D\right]}_{O_{A}}^{O_{B}}{\left[R\right]}_{O_{A}}^{O_{B}} & {\left[R\right]}_{O_{A}}^{O_{B}} \end{array}\right]$$

where [***R***${]}_{OA}^{OB}$ is the coordinate-transformation rotation matrix. [***D***${]}_{OA}^{OB}$ is defined by the translation vector [***d***${]}_{OA}^{OB}$ = [*x*,*y*,*z*]*^T^*, which takes the following form:

$${\left[D\right]}_{C_{1}}^{C}=\left[\begin{array}{ccc} 0 & -z & y\\ z & 0 & -x\\ -y & x & 0 \end{array}\right]$$
